# Effect of Topical Intra-Auricular Lidocaine on Tinnitus: A Randomized Double-Blind Placebo-Controlled Study

**DOI:** 10.1055/s-0045-1810028

**Published:** 2025-10-16

**Authors:** Tatiane Vacaro Campos, Ricardo Ferreira Bento, Jeanne Oticica, Maíra Said Jabour, Nathália Távora, Isabela Jardim, Cristiane Leite de Marchi, Robinson Koji Tsuji

**Affiliations:** 1 Department of Ophthalmology and Otorhinolaryngology, Faculty of Medicine, Hospital das Clínicas da Universidade de São Paulo, São Paulo, SP, Brazil; 2 Department of Otorhinolaryngology, Hospital Universitário Cassiano Antônio, Universidade Federal do Espírito Santo, Vitória, ES, Brazil; 3 Centro de Audiologia (Audiocenter), São Paulo, SP, Pinheiros, Brazil

**Keywords:** tinnitus, lidocaine, external acoustic meatus, local anesthetics, topical administration, clinical trial

## Abstract

**Introduction:**

Tinnitus is a prevalent symptom and has no proven effective treatment. Clinical observations that some patients reported significantly improved tinnitus after topical ear drop administration of lidocaine motivated research on this drug.

**Objective:**

Evaluate the action of topical intra-auricular 10% lidocaine in suppressing tinnitus compared to placebo.

**Methods:**

This was a randomized, crossover, double-blind, placebo-controlled clinical trial. The variations of the visual analog scale (VAS), tinnitus loudness, and the minimum masking level (MML) after intra-auricular lidocaine 10% were compared with a placebo. The same variables were compared with some sample features.

**Results:**

There was no statistically significant difference between the action of lidocaine and placebo in tinnitus variation measured by VAS (p = 0.361), tinnitus loudness (p = 0.850), and MML. (p = 0.213). The VAS variation was statistically lower in patients with any hearing loss (p = 0.005); the tinnitus loudness variation was higher in patients with tinnitus modulation (p = 0.049) and lower in patients with any hearing loss (p = 0.045). The MML variation was lower in patients with bruxism/TMD (p = 0.026).

**Conclusion:**

In this study the action of lidocaine on the variation of tinnitus intensity was similar to placebo. We believe that the use of manipulated medication may have interfered in the outcome and that the large number of patients with positive response to placebo should be investigated in further studies.

## Introduction

Subjective tinnitus is a symptom related to the conscious perception of an auditory sensation without an external sound stimulus, 1 thus being perceived exclusively by the patient. 2 3

It is a common condition caused by an abnormal central auditory pathway activity, usually secondary to a lesion in the peripheral auditory pathway. 4 5 Its prevalence has been estimated between 8.2–30.3% in industrialized countries, 6 and it can be disabling in 1–3% of bothersome tinnitus cases. 7 For many years, tinnitus was thought to be the result of abnormal neural activity unique to the auditory pathways. An increasing number of studies have suggested a much more complex and multimodal mechanism involving the somatosensory system, 8 9 10 11 the limbic system, 12 and the autonomic nervous system, 13 in addition to the auditory pathway. Neuroimaging research confirmed the involvement of mechanisms related to memory and cognition in the perception, persistence, anxiety, distress, and suffering associated with tinnitus. 14 15

Although many drugs have been tested for tinnitus treatment, there is still no specific and broadly effective medication. 1 Research with intravenous (IV) lidocaine demonstrated tinnitus suppression rates ranging from 40–80%. 16 Nevertheless, its effect is usually transient, and there is a risk of toxicity at high doses. Lidocaine is a local anesthetic, antiarrhythmic, and membrane stabilizer that acts on voltage-dependent ion channels, in addition to influencing glutamate, gamma-aminobutyric acid, glycine, and vanilloid receptors. 17 It acts primarily on sodium channels on the inner surface of nerve cell membranes, preventing neural depolarization. 18

To avoid the risk of side effects of IV lidocaine, some studies have investigated its action on tinnitus using other routes of administration, such as intratympanic, 19 intradermal, 20 and transdermal, 21 finding variable results, not always free of side effects. To date, we have found no studies evaluating the action of lidocaine on tinnitus when applied topically intra-auricularly.

The lack of safe, non-invasive, effective, and inexpensive treatment to provide long-term relief is frustrating for both the patient and the physician.

The motivation for this study was the information, brought spontaneously by some patients, about the perception of reduced chronic tinnitus intensity after using ear drops that were primarily indicated for the treatment of otitis externa. We therefore designed this study with the aim of evaluating the action of topical intra-auricular 10% lidocaine in suppressing tinnitus compared to a placebo, which has the advantages of avoiding the adverse effects of systemic lidocaine administration and being easily used and cost-effective.

## Methods

### Trial Design

This project was designed as a randomized, crossover, placebo-controlled, double-blind, clinical trial. It was approved by the Research Ethics Committee (REC) of the host institution under opinions number 4.188.11 and 5.562.868 and was registered at ClinicalTrial.gov under identification number NCT05711641. The participants were properly informed and signed an informed consent form.

### Participants

- Eligibility criteria: The inclusion criteria were age over 18 years, continuous tinnitus for at least six months, and a visual analog scale (VAS) tinnitus intensity score of at least three points on the days the substances were administered. The exclusion criteria were otologic infection, tympanic membrane perforation, anatomic external ear changes, pulsatile tinnitus, objective tinnitus, known allergy to lidocaine or other topical anesthetics, pregnancy, cardiac arrhythmia, epilepsy, previous history of seizure, or use of anticonvulsants.
- Settings and locations where the data were collected: The study participants were selected among patients who sought care at an otorhinolaryngology center specializing in tinnitus, in the period between April 7, 2021, and April 6, 2022. After analyzing the medical records, 113 patients met the inclusion criteria, and none of the exclusion criteria.

### Interventions

Patients were evaluated on two visits 15 days apart. On the first day, they underwent a complete clinical evaluation, including medical history, otorhinolaryngologic examination, somatosensory tinnitus testing, and audiological evaluation, as well as the study-specific tests consisting of tinnitus intensity measurement by VAS and psychoacoustic measures. On the second day, only the specific tests were performed.

The audiological evaluation was carried out in an acoustic treatment booth, with TDH 39 and HD 200 headphones, using the MADSEN Itera II audiometer. The tonal threshold between 250–16 kHz was researched by an ascending and descending technique. A tonal threshold up to 8 kHz was classified according to the criteria by Lloyd and Kaplan. 22 As tinnitus can be related to any degree of alteration in hearing function, including unidentified losses, we registered patients who had a tonal threshold greater than 25dBHL in at least one frequency up to 8000Hz, which we called “any hearing loss”. The audiometric thresholds of high frequencies (9–16 kHz) were measured to determine the tinnitus pitch in these frequency ranges.

The psychoacoustic measures used for analysis in this study were the tinnitus loudness and the minimum masking level (MML), measured in decibel sensation level (dBSL) and performed before and after the application of each of the substances. The pitch was assessed by pure tone sound stimuli or narrow band (NB) noise, being measured in Hz.

Tinnitus loudness was analyzed by presenting the sound most similar to the patient's tinnitus, determined by the pitch, in the ipsilateral ear in cases of unilateral tinnitus or the ear with better hearing in cases of bilateral tinnitus, increasing the intensity by 1 dB until finding the volume closest to the patient's tinnitus.

The MML was assessed by an NB sound presented to the ipsilateral ear. Starting from the patient's threshold detected for this type of sound, the volume was increased by 1 dB every four seconds until the presented sound masked the tinnitus.

The VAS of tinnitus intensity, ranging from 0 to 10 points, was measured as soon as the patient was positioned to receive the medication and immediately after the medication was completely removed from the ear canal after 5 minutes. VAS was also used to evaluate somatic maneuvers to determine tinnitus variation (called modulation), which was considered positive if there was a change in VAS score in at least one of the maneuvers.

The substances used were 10% lidocaine (active substance) or distilled water (placebo). They were prepared in the same compounding pharmacy, replaced every 90 days, and stored in identical bottles identified as: "Substance 1" and "Substance 2." The preference for the use of manipulated lidocaine over the commercial product is because it avoids the presence of other substances, such as preservatives, that could interfere with the test results.

The patient was positioned in a lateral decubitus position, with the tested ear facing up, and 20 drops of the randomized substance were administered and remained in the external acoustic meatus (EAM) for five minutes at each treatment session. For medication removal, the patient was instructed to sit with their head tilted to the side of the study ear, the substance was removed by gravity, and the ear was dried with gauze.

Each ear was its own control and crossover administration was two weeks apart. The washout time was determined considering that the half-life of lidocaine is approximately 2h and may reach 3.5h in cases of patients with severe liver disease. 23

### Outcomes

- Primary Outcomes: Comparison of the action of lidocaine vs. placebo on the variation of the averages of VAS, tinnitus loudness, and MML
- Secondary Outcomes: To investigate whether any characteristics of the sample correlated with tinnitus variation after the administration of the substances, we compared the VAS, tinnitus loudness *,* and MML variations related to the variables sex, bruxism/ temporomandibular disorder (TMD), cervical changes, somatosensory tinnitus, modulation, and hearing loss.

### Sample Size

The VAS of tinnitus intensity was chosen as the basis for calculating the sample since it is one of the most widely used measures in tinnitus research. Based on the goal of reducing the VAS score of tinnitus intensity after the use of topical lidocaine compared to placebo in the same ear, assuming that the VAS score for tinnitus would be reduced by an average of 2 points more with the use of lidocaine than with placebo, considering variability between patients of 2.6 points in the reduction of the (SD = 2.6 points), with 80% power and 95% confidence the sample required for the study was 27 patients, considering a two-tailed test. Considering a 20% loss to follow-up, the final number of participants was calculated at 33 patients.

**Fig. 1:**
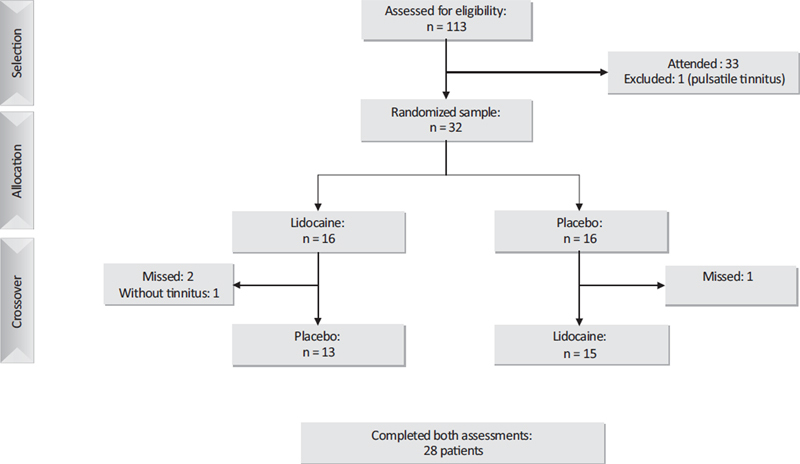
Flowchart showing the methodology and the number of participants in each stage of the study.

The patients were invited to take part in the study via WhatsApp. Of the 33 patients who responded and showed up, one was excluded for having only pulsatile tinnitus on the day of the appointment, and 28 completed both assessments ( Fig. 1 ).

### Randomization

The website www.randomization.com was used to define the order of substance administration, defining whether the patient would start the study as a case or a control. The subjects were randomized by the “Heads or Tails” method in cases of bilateral and symmetrical tinnitus, with one Real coin (the official currency of the country) being tossed by the patient himself. “Heads” was previously defined as the right ear, and “tails” as the left ear. In the case of bilateral and asymmetrical tinnitus, the study ear was the ear where the tinnitus was perceived with greater intensity.

### Blinding

The study was blinded to both the patient and the professional who applied the substances and collected the data.

### Statistical Analysis

Qualitative characteristics of patients and tinnitus were described using absolute and relative frequencies, and quantitative characteristics were described using summary measures (mean, standard deviation, median, minimum, and maximum value). 24 VAS, tinnitus loudness, MML, and variations for each administration were described using summary measures, and the variations between substances were compared using Generalized Estimating Equations (GEE) with marginal normal distribution and identity link function, assuming an interchangeable correlation matrix between substances. 25

The action of lidocaine and placebo on the study variables were compared between the patient and tinnitus characteristics described above, using the same type of two-factor analysis: substance and characteristic of interest. The results were analyzed by Bonferroni's multiple comparisons to identify between which substances or characteristics the differences occurred. 26

The analyses were performed using the IBM-SPSS for Windows version 22.0 software and tabulated using the Microsoft Excel 2010 software. Statistical significance was set at 5%.

## Results

A total of 32 patients were evaluated. The general characteristics of the sample are described in Table 1 . The study ear presented hearing loss in 18.8% of the sample, but 20 of the 26 patients with audiological thresholds classified as normal had a tone threshold higher than 25 dB hearing level (dBHL) in at least one frequency up to 8,000 Hz, which we called any hearing loss.

**Table 1:** Sample data regarding age, gender, degree of impact of tinnitus on quality of life, laterality of the study ear, clinical comorbidities and the presence of hearing loss

Variable	Description
	(n = 32)
**Age (years)**	
Mean ± SD	54 ± 13.7
Median (min.; max.)	57.5 (21; 78)
**Sex, n (%)**	
Male	18 (56.3)
Female	14 (43.8)
**Total THI**	
Mean ± SD	31.4 ± 17.1
Median (min.; max.)	31 (10; 84)
**Study ear, n (%)**	
Right	12 (37.5)
Left	20 (62.5)
**Comorbidities, n (%)**	
Presence of pain	13 (40.6)
Dizziness	6 (18.8)
DM	4 (12.5)
SAH	10 (31.3)
Dyslipidemia	14 (43.8)
Hypothyroidism	2 (6.3)
Anxiety/Depression	13 (40.6)
Cervical spine dysfunction	15 (46.9)
Bruxism/TMD	18 (56.3)
**Classification of auditory thresholds (study ear), n (%)**	
Normal	26 (81.3)
Mild	2 (6.3)
Moderate	2 (6.3)
Moderataly severe	2 (6.3)
**Presence of hearing loss in the contralateral ear**	
Lloyd e Kaplan, n (%)	7 (21.9)

Abbreviations: DM, diabetes mellitus; max., maximum; min., minimum; SAH, systemic arterial hypertension; SD, standard deviation; TMD, temporomandibular dysfunction.

**Fig. 2:**
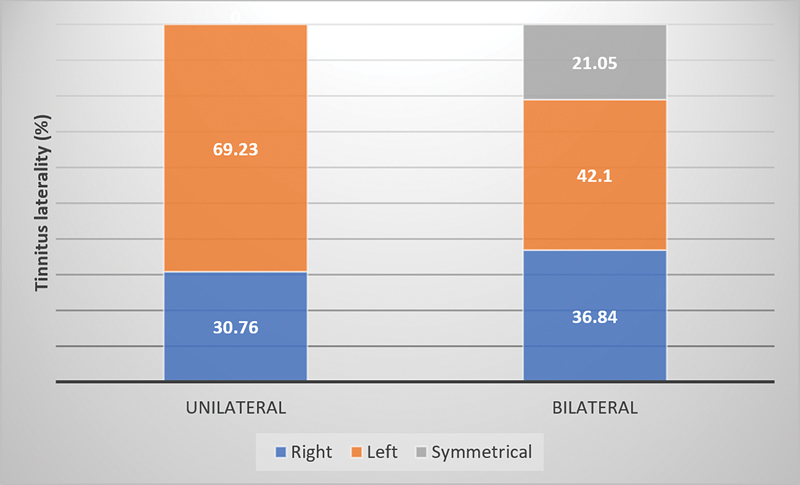
Tinnitus location in relation to being uni or bilateral and in relation to symmetry.

The mean time of tinnitus perception was 70.9 (±69.2) months in the study ear. The most frequent type of tinnitus was whistling, with 21 patients presenting only one type of tinnitus and the others presenting two types. Somatosensory tinnitus was diagnosed with 43.8% (14) of patients and in 59.4% (19) there was a variation in tinnitus characteristics after somatic testing. Figure 2 presents the characteristics of tinnitus about its location.

Pure tone was the most common type of tinnitus, being mostly referred to at the frequency of 8,000 Hz. The acuphenometry results are shown in Supplementary File 1 (Acuphenometry results before and after lidocaine or placebo in the 32 patients).

The mean pre-medication VAS was compared between the first and second evaluation days to verify tinnitus stability. The results mean VAS of 6.47 and 6.18 points, respectively on the first and second days, showing no significant variation between dates that could interfere with test results.

There was also no difference between the mean premedication MML of the first day (4.5 dBNS) and the second day (4.54 dBNS) and between the mean premedication of tinnitus loudness on the first day (6.71dBNS) and the second day (6.75dBNS).

**Fig. 3:**
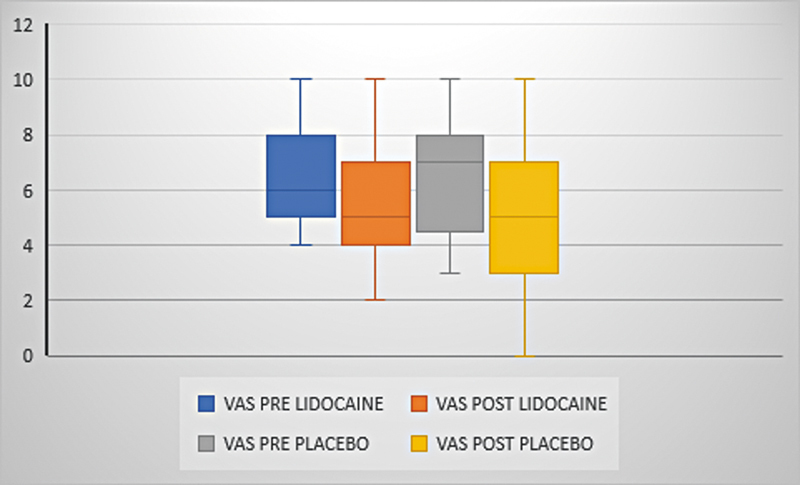
VAS variation. Presentation of the mean, median, minimum and maximum values of the VAS. Comparison between pre and post medication times (p = 0.361). VAS: visual analog scale.

**Fig. 4:**
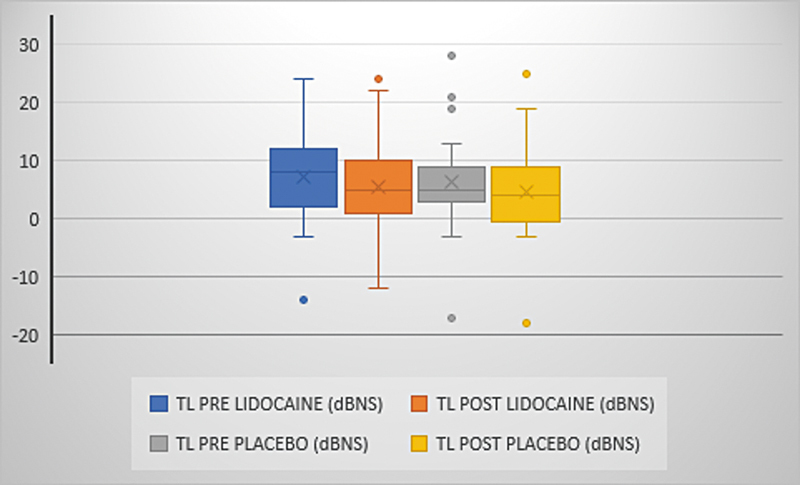
Tinnitus loudness variation. Presentation of the mean, median, minimum and maximum values and outliers of tinnitus loudness. Comparison between pre- and post-medication times (p = 0.850). TL: tinnitus loudness.

**Fig. 5:**
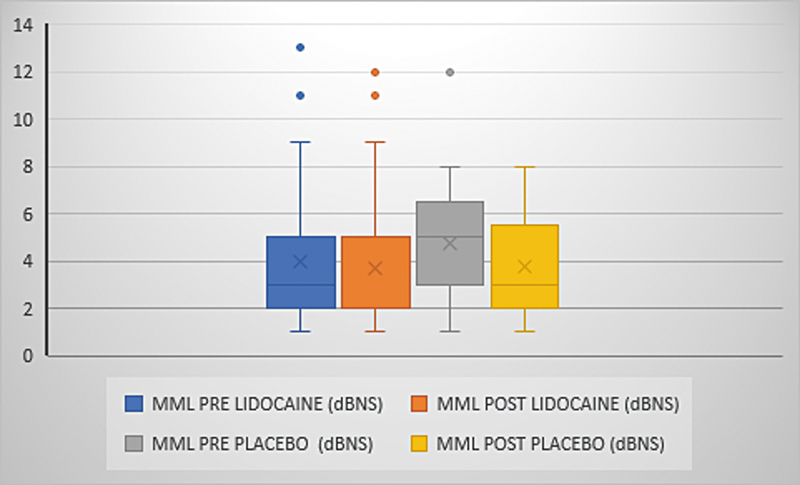
MML variation. Presentation of the mean, median, minimum and maximum values and outliers of the MML. Comparison between pre- and post-medication times (p = 0.213). MML: minimum masking level.

The mean VAS ranged from 6.3 (±1.8) to 5.5 (±2.1) with lidocaine and from 6.3 (±2) to 5.2 (±2.6) with placebo (p = 0.361) ( Fig. 3 ). The mean tinnitus loudness ranged from 7.2 (±7.8) to 5.6 (±7.7) with lidocaine and 6.4 (±8.4) to 4.6 (±8.3) with placebo (p = 0,850) and the mean MML ranged from 4 (±3) to 3.7 (±2.9) with lidocaine and from 4.8 (±2.4) to 3.8 (±2.2) with placebo (p = 0.213) ( Figs. 4 and 5 ).

When the variation in VAS, tinnitus loudness and MML were compared with the characteristics of the sample, the following results were found: the mean VAS variation was statistically lower in patients with any hearing loss, regardless of the substance used (p = 0.005); the mean tinnitus loudness variation was statistically higher in patients with tinnitus modulation, regardless of the substance use (p = 0.049), being lower in patients with any hearing loss, also regardless of the substance used (p = 0.045); the mean MML variation was statistically lower in bruxism/ TMD patients, regardless of the substance used (p = 0.026). Table 2 . Description of the average VAS variation according to substances and characteristics of interest; the result of the comparative analyses. Table 3 . Description of the average Tinnitus Loudness variation according to substances and characteristics of interest; the result of the comparative analyses. Table 4 . Description of the average MML variation according to substances and characteristics of interest; the result of the comparative analyses).

**Table 2:** Description of the average VAS variation according to substances and characteristics of interest; result of the comparative analyses

Factor	Lidocaine		Placebo		p _Substance_	p _Factor_	p _Interaction_
Sex	Male	Female	Male	Female	0.352	0.893	0.689
mean ± SD	-0.9 ± 1.1	-0.7 ± 1.1	-1.1 ± 1.5	-1.2 ± 1.4			
median (min.; max.)	0 (-3; 0)	0 (-3; 0)	0 (-4; 0)	-0.5 (-3; 0)			
**Bruxism/TMD**	**No**	**Yes**	**No**	**Yes**	0.317	0.256	0.174
mean ± SD	-0.7 ± 1	-0.9 ± 1.2	-1.6 ± 1.6	-0.7 ± 1.2			
median (min.; max.)	0 (-3; 0)	0 (-3; 0)	-1.5 (-4; 0)	0 (-3; 0)			
**Cervical Spine Disfunction**	**No**	**Yes**	**No**	**Yes**	0.378	0.232	0.675
mean ± SD	-0.6 ± 1	-1.1 ± 1.2	-1.1 ± 1.4	-1.2 ± 1.5			
median (min.; max.)	0 (-3; 0)	-1 (-3; 0)	0 (-4; 0)	0 (-4; 0)			
**Somatosensory tinnitus**	**No**	**Yes**	**No**	**Yes**	0.429	0.276	0.250
mean ± SD	-0.5 ± 0.9	-1.2 ± 1.1	-1.2 ± 1.6	-1.1 ± 1.3			
median (min.; max.)	0 (-3; 0)	-1 (-3; 0)	0 (-4; 0)	0 (-3; 0)			
**Modulation**	**No**	**Yes**	**No**	**Yes**	0.239	0.520	0.130
mean ± SD	-0.4 ± 1	-1.1 ± 1.1	-1.4 ± 1.7	-1 ± 1.3			
median (min.; max.)	0 (-3; 0)	-1 (-3; 0)	0 (-4; 0)	0 (-3; 0)			
**Hearing loss in the study ear** **(any up to 8Kz)**	**No**	**Yes**	**No**	**Yes**	0.491	**0.005**	0.987
mean ± SD	-1.5 ± 1.2	-0.6 ± 1	-1.8 ± 1.5	-1 ± 1.4			
median (min.; max.)	-2 (-3; 0)	0 (-3; 0)	-2.5 (-3; 0)	0 (-4; 0)			
**Haring loss in the study ear** **(Lloyd e Kaplan)**	**No**	**Yes**	**No**	**Yes**	0.138	0.965	0.219
mean ± SD	-0.9 ± 1.1	-0.3 ± 0.8	-1 ± 1.3	-1.6 ± 2.2			
median (min.; max.)	0 (-3; 0)	0 (-2; 0)	0 (-3; 0)	0 (-4; 0)			

Abbreviations: max, maximum; min, minimum; SD, standar deviation.

EEG with normal distribution and identity linkage function with interchangeable correlation between substance.

**Table 3:** Description of the average Tinnitus Loudness variation according to substances and characteristics of interest; result of the comparative analyses

Factor	Lidocaine		Placebo		p _Substance_	p _Factor_	p _Interaction_
Sex	Male	Female	Male	Female	0.892	0.163	0.962
mean ± SD	-1.1 ± 2.7	-2.3 ± 3.5	-1.1 ± 3.1	-2.4 ± 3.5			
median (min.; max.)	-2 (-5; 5)	-1 (-11; 2)	0 (-8; 4)	-2.5 (-9; 5)			
**Bruxism/TMD**	**No**	**Yes**	**No**	**Yes**	0.805	0.806	0.385
mean ± SD	-1.1 ± 3.4	-2.1 ± 2.8	-2 ± 4	-1.5 ± 2.6			
median (min.; max.)	0 (-11; 3)	-2 (-7; 5)	-1.5 (-9; 5)	0 (-8; 1)			
**Cervical spine disfunction**	**No**	**Yes**	**No**	**Yes**	0.867	0.433	0.158
mean ± SD	-0.8 ± 3.2	-2.5 ± 2.8	-1.9 ± 4.1	-1.6 ± 2.3			
median (min.; max.)	0 (-11; 5)	-3 (-7; 3)	0 (-9; 5)	-1 (-7; 1)			
**Somatosensory tinnitus**	**No**	**Yes**	**No**	**Yes**	0.924	0.584	0.272
mean ± SD	-1.1 ± 3.2	-2.4 ± 2.9	-1.9 ± 4	-1.6 ± 2.4			
median (min.; max.)	0 (-11; 3)	-2.5 (-7; 5)	-1 (-9; 5)	0 (-7; 1)			
**Modulation**	**No**	**Yes**	**No**	**Yes**	0.869	**0.049**	0.665
mean ± SD	-0.5 ± 2.2	-2.5 ± 3.4	-0.9 ± 3.6	-2.3 ± 3.2			
median (min.; max.)	0 (-5; 3)	-2 (-11; 5)	-1 (-7; 5)	-0.5 (-9; 1)			
**Hearing loss in the study ear** **(any up to 8Kz)**	**No**	**Yes**	**No**	**Yes**	0.513	**0.045**	0.194
mean ± SD	-4.3 ± 3.7	-1 ± 2.6	-2.5 ± 2	-1.6 ± 3.6			
median (min.; max.)	-3.5 (-11; 0)	-1 (-7; 5)	-3.5 (-4; 0)	0 (-9; 5)			
**Hearing loss in the study ear** **(Lloyd e Kaplan)**	**No**	**Yes**	**No**	**Yes**	0.922	0.100	0.992
mean ± SD	-2 ± 3.1	-0.2 ± 2.6	-2.1 ± 3	-0.2 ± 4.9			
median (min.; max.)	-2 (-11; 5)	0 (-5; 2)	-0.5 (-9; 1)	-1 (-7; 5)			

EEG with normal distribution and identity linkage function with interchangeable correlation between substance. SD: standard deviation; min: minimum; max: maximum.

**Table 4:** Description of the average MML variation according to substances and characteristics of interest; result of the comparative analyses

Factor	Lidocaine		Placebo		p _Substance_	p _Factor_	p _Interaction_
Sex	Male	Female	Male	Female	0.247	0.902	0.367
mean ± SD	-0.1 ± 1.4	-0.6 ± 1.8	-1.2 ± 2.8	-0.8 ± 1.6			
median (min.; max.)	0 (-3; 2)	0 (-5; 1)	0 (-10; 1)	-1 (-4; 2)			
**Bruxism/TMD**	**No**	**Yes**	**No**	**Yes**	0.246	**0.026**	0.989
mean ± SD	-0.9 ± 2	0.1 ± 1.2	-1.5 ± 2.9	-0.5 ± 1.4			
median (mn.; max.)	-0.5 (-5; 2)	0 (-2; 2)	-1 (-10; 1)	0 (-3; 2)			
**Cervical Spine Disfunction**	**No**	**Yes**	**No**	**Yes**	0.223	0.101	0.747
mean ± SD	0.1 ± 1.4	-0.8 ± 1.7	-0.7 ± 1.5	-1.3 ± 2.9			
mediana (mín.; máx.)	0 (-3; 2)	0 (-5; 1)	0 (-4; 1)	-1 (-10; 2)			
**Somatosensory tinnitus**	**No**	**Yes**	**No**	**Yes**	0.226	0.760	0.961
mean ± SD	-0.2 ± 1.6	-0.4 ± 1.7	-0.9 ± 1.4	-1.1 ± 3			
median (min.; max.)	0 (-3; 2)	0 (-5; 1)	-1 (-4; 1)	0 (-10; 2)			
**Modulation**	**No**	**Yes**	**No**	**Yes**	0.197	0.867	0.651
mean ± SD	-0.2 ± 1.7	-0.4 ± 1.6	-1.2 ± 1.5	-0.9 ± 2.6			
median (min.; max.)	0 (-3; 2)	0 (-5; 2)	-1 (-4; 1)	0 (-10; 2)			
**Hearing loss in the study ear** **(any up to 8Kz)**	**No**	**Yes**	**No**	**Yes**	0.733	0.603	0.287
mean ± SD	-1.2 ± 2.4	-0.1 ± 1.4	-0.7 ± 2.1	-1.1 ± 2.3			
median (min.; max.)	-0.5 (-5; 1)	0 (-3; 2)	-0.5 (-3; 2)	-1 (-10; 1)			
**Hearing loss in the study ear** **(Lloyd e Kaplan)**	**No**	**Yes**	**No**	**Yes**	0.486	0.414	0.715
mean ± SD	-0.4 ± 1.6	-0.2 ± 1.7	-1.1 ± 2.4	-0.4 ± 1.7			
median (min.; max.)	0 (-5; 2)	0 (-3; 2)	-1 (-10; 2)	0 (-3; 1)			

Abbreviations: max, maximum; min, minimum; SD, standard deviation.

EEG with normal distribution and identity linkage function with interchangeable correlation between substances.

## Discussion

The conscious perception of an auditory sensation without an external sound stimulus is called tinnitus, a complex and heterogeneous symptom regarding diagnosis, the impact on quality of life, and treatment.

This symptom can be catastrophic in up to 12.7% of cases associated with disorders such as anxiety and depression. 27 In our sample there was a high prevalence of symptoms of anxiety or depression, but the degree of tinnitus annoyance was classified as mild in the Tinnitus Handicap Inventory (THI) in most individuals.

Many substances have been investigated in the search for a medication that acts effectively on tinnitus and can be used as a treatment, including lidocaine. 28 The action of intravenous lidocaine is the most widely studied, with success rates up to 80%, 16 but always with a fleeting effect and potential risks for significant adverse events, 29 which made its therapeutic use unfeasible. Other researchers investigated the intratympanic pathway, 19 with variable responses but with patients experiencing a lot of dizziness. More recently, intradermal injections 20 into the EAM have been evaluated with no side effects.

Knowing that lidocaine has a suppressive effect on the perception of tinnitus and motivated by reports of improvement in this symptom from some patients who had used ear drops containing 5% lidocaine, neomycin, and hyaluronidase, originally prescribed for treating otitis externa, we designed this study with the hypothesis that lidocaine applied topically in the ear could have an effect on the perception of tinnitus.

To date, we haven't found any articles in the literature that have evaluated the action of topically applied lidocaine on tinnitus intensity. This demonstrates the original nature of this research, but on the other hand, it prevents us from comparing our results with those of similar studies. The only studies that investigated the action of non-injectable lidocaine on tinnitus intensity were those related to iontophoresis 30 31 and those that evaluated the use of lidocaine patches. 21 Iontophoresis was used to favor the penetration of lidocaine into the middle ear and the results ranged from 4% to 62% improvement in tinnitus, according to a recent literature review whose conclusion was that, due to the heterogeneity and low quality of studies, there is no clarity regarding the results. 31 In general, these studies used 2% lidocaine, 31 therefore a much lower concentration than that used in our study. Regarding the study with lidocaine patches, although the results were positive, few patients followed the treatment until the end due to side effects and treatment costs. Compared to the study by O'Brien et al., 2019, 21 our research has the advantage of low cost in addition to the fact that none of the patients had side effects, but the medication was in contact with the skin for a shorter time.

The variables studied in this research (VAS, tinnitus loudness, and MML) showed no statistically significant differences in mean variations after the topical intra-auricular administration of lidocaine or placebo. The factors associated with this result may be related to the small sample size, despite reaching the minimum number stipulated in sample calculation, the use of manipulated medication, and the time of substance administration.

We chose to use 10% lidocaine manipulated in a distilled water vehicle to avoid any bias in determining the substance responsible for tinnitus reduction since the corresponding commercial formulation contains more excipients than the lidocaine used in IV studies. The 2% lidocaine for general IV use contains sodium chloride, methylparaben, sodium hydroxide, and water for injection (package insert, 2% Xylestesin® with no vasoconstrictor), 32 while the commercial 10% spray formulation contains saccharin sodium, disodium edetate, propylene glycol, cherry/mint flavor, ethyl alcohol, and purified water (package insert, 10% Xylestesin® spray). 33 Excipients are substances considered pharmacologically inactive, which complete the mass or volume of a drug. Although they are generally used only as vehicles, they can modify the physicochemical properties and bioavailability of a substance. 34 Furthermore, studies on the action of excipients such as propylene glycol on the metabolism of orally used drugs concluded that these substances, despite being considered pharmacologically inert, can change the pharmacokinetic properties of the active ingredient by several mechanisms, including hepatic enzyme inhibition. 35 Another factor associated with the permeation of drugs into the skin is the presence of alcohol in their formulation. Solvents can remove lipids from the corneal extract, reducing the barrier function, 36 therefore the absence of alcohol in the manipulated formulation may have negatively interfered with the absorption of lidocaine.

Interestingly, our results showed that 11 of 29 patients had reduced VAS intensity of at least two points after placebo administration, and two of these patients (numbers 2 and 24) achieved complete tinnitus suppression. Studies comparing IV lidocaine and placebo showed no responses with placebo (saline solution), 37 which differed from this study and from studies on intra-auricular lidocaine and iontophoresis, 38 which highlighted the possibility of some mechanical effect on the tympanic membrane changing the middle ear pressure, which would be transferred to the inner ear, improving the tinnitus. Specific studies comparing patients with and without middle ear pressure changes could elucidate this question.

Since some patients showed tinnitus reduction either with lidocaine or placebo, we compared the mean variation in study variables regarding some sample characteristics and found that the tinnitus loudness variation was higher in patients with modulation and lower in patients with any hearing loss. This information suggests that patients with muscular or osteoarticular disorders in regions near the ear could benefit from the local action of lidocaine on nerve endings, as in the study by Vielsmeier et al., who reported improvement in patients with signs of somatosensory tinnitus with the anesthesia of trigeminal structures using intraoral injectable lidocaine in the region of the optic ganglion. 39 On the other hand, this hypothesis has no support because there was no association with the variable somatosensory tinnitus, and the variable bruxism/TMD was negatively correlated with MML, contrary to what was expected.

The site of action of lidocaine on the auditory pathway was extensively researched, with some authors reporting a peripheral action 40 and others considering a central action. 41 To investigate this question, we chose to evaluate only one ear even in cases of bilateral tinnitus, if a systemic effect should generate tinnitus changes in both ears. No patient showed a response in the contralateral ear, even those who responded in the study ear, suggesting that the action must occur in the peripheral auditory pathway ipsilateral to the studied side. Another piece of information supporting this hypothesis is the fact that, in addition to showing no response in the contralateral ear, none of our patients showed systemic absorption symptoms like those found in IV use. 18

Considering that there is an effect of topical lidocaine on tinnitus that could not be proven in this study and that is not systemic, at which site could lidocaine be acting? According to Majumdar et al. (1983), 42 pharmacokinetic studies showed that IV lidocaine is rapidly distributed in the blood into well-vascularized tissues; therefore, its action is almost immediately in these areas. The drug is then distributed to less vascularized tissues. The same may occur with topical administration; therefore, the medication would act first on the tympanic membrane and may or may not be absorbed into the middle ear. Considering that the medication could pass the tympanic membrane and reach the middle ear and windows, none of the patients described any type of dizziness, with vertigo being one of the most frequent adverse effects reported in studies on intratympanic lidocaine.

In the present study, besides the absence of adverse effects, two patients described a sensation of hearing improvement, regardless of tinnitus improvement, differently from what is reported in the literature in cases of intratympanic administration, which can result in usually mild transient sensorineural deafness at low frequencies. 43

Finally, we believe that the strengths of our study were the randomized, double-blind, placebo-controlled design, safety, and low cost.

Although we found no statistically significant difference between lidocaine and placebo on tinnitus, new studies should be carried out with topical intra-auricular medications due to the positive responses found with both substances in some patients. Not using commercial 10% lidocaine may have interfered with the absorption of the medication by the EAM, and larger samples may give responses related to specific characteristics that can guide us to more appropriate studies.

## Conclusion

This study showed no statistically significant difference between the action of topical intra-auricular lidocaine and placebo on tinnitus intensity.
