# Supplementary material for: Effect of Topical Intra-Auricular Lidocaine on Tinnitus: A Randomized Double-Blind Placebo-Controlled Study
Source: Int Arch Otorhinolaryngol. 2025 Oct 16;29(4):1–11. doi: 10.1055/s-0045-1810028 (PMC12530916; doi:10.1055/s-0045-1810028)
Supplement: Supplementary file 1 — Supplementary Material [file 10-1055-s-0045-1810028-s231635.pdf]

Suplemental File 1 Acuphenometry results before and after lidocaine or placebo in the 32 patients

Patient	Tinnitus types (study ear) Day 1 / Day 2	Pitch pre lidocaíne Hz	Pitch post lidocaíne Hz	Loudness pre lidocaíne dBNS	Loudness post lidacaíne dBNS
1	Pure tone	12500	12500	20	21
2	Pure tone	6000	8000	14	3
3	Pure tone	12500	12500	9	6
4	Pure tone	10000	10000	8	6
5	Pure tone	6000	6000	9	4
6	Pure tone	1000	1000	34	34
7	Warble	8000	8000	8	8
8	Pure tone	9000	9000	7	6
9	Pure tone	8000	8000	−3	−3
10	Pure tone	8000	8000	2	2
11	Pure tone	8000	8000	5	3
12	Pure tone	8000	8000	13	10
13	Warble/Pure tone	8000	8000	2	1
14	Pure tone	6000	6000	9	9
15	Pure tone	8000	8000	3	6
16	Pure tone	6000	6000	0	2
17	Pure tone/Narrow band	6000	6000	1	1
18	Pure tone	750	750	13	13
19	Warble pre placebo, Pure tone post placebo / Pure tone	8000	8000	13	11
20	Pure tone	Missed	Missed	Missed	Missed
21	Pure tone / Narrow band	250	250	24	22
22	Narrow band	12500	12500	2	0
23	Speech noise	Speach noise	Speech noise	12	5
24	Narrow band	8000	8000	−14	−12
25	Narrow band / Speech noise	Speech noise	Speech noise	3	−1
26	Pure tone	9000	8000	8	13
27	Narrow band	9000	9000	2	−3
28	Pure tone	9000	9000	3	−2
29	Narrow band	8000	8000	0	0
30	Pure tone	8000	8000	10	7
31	Pure tone	9000	8000	5	1
32	Pure tone	6000	6000	10	10

 © 2025. The Author(s).

Eletronic supplementary material – Acuphenometry results before and after lidocaine or placebo in the 32 patients (continued).

Patient	MML pre lidocaíne dBNS	MML post lidocaíne dBNS	Pitch pre placebo Hz	Pitch post placebo Hz	Loudness pre placebo dBNS	Loudness post placebo dBNS	MML pre placedo dBNS	MML post placebo dBNS
1	2	2	Missed	Missed	Missed	Missed	Missed	Missed
2	3	2	8000HZ	8000HZ	3	−1	1	1
3	11	12	11200	11200	28	25	1	2
4	1	1	6000	60000	19	19	7	7
5	1	2	9000	9000	3	−1	4	1
6	6	7	1000	1000	21	13	7	6
7	1	1	8000	8000	4	4	1	1
8	4	2	9000	8000	6	−3	3	1
9	4	5	6000	6000	8	8	7	3
10	9	9	9000	8000	3	2	3	2
11	2	2	8000	8000	5	−2	1	1
12	7	2	6000	6000	6	2	5	6
13	3	4	11200	12500	5	0	4	3
14	3	3	4000	4000	10	10	8	8
15	4	2	8000	8000	0	2	6	4
16	3	4	8000	8000	5	−2	3	4
17	2	2	750	750	6	4	5	2
18	1	3	1000	1500	12	16	3	4
19	2	1	8000	8000	20	16	5	3
20	Missed	Missed	1000	2000	5	6	3	4
21	13	11	6000	6000	4	4	7	8
22	2	4	Missed	Missed	Missed	Missed	Missed	Missed
23	4	5	Speech noise	Speech noise	7	6	5	5
24	3	2	8000	8000	−17	−16	3	3
25	1	2	3000	3000	0	−3	6	3
26	4	4	8000	8000	4	4	3	3
27	3	2	9000	9000	−1	0	5	4
28	2	2	Without tinnitus	Without tinnitus	Without tinnitus	Without tinnitus	Without tinnitus	Without tinnitus
29	6	3	9000	8000	−3	2	4	3
30	9	6	8000	8000	5	5	8	7
31	3	1	9000	9000	4	4	4	3
32	5	5	6000	6000	13	13	5	7

 © 2025. The Author(s).
